# Chemisorption and sustained release of cefotaxime between a layered double hydroxide and polyvinyl alcohol nanofibers for enhanced efficacy against second degree burn wound infection†

**DOI:** 10.1039/c9ra08355c

**Published:** 2020-04-01

**Authors:** Maha B. Abd Elhaleem, Ahmed A. Farghali, Ahmed. A. G. El-Shahawy, Fatma I. Abo El-Ela, Zienab E. Eldine, Rehab Khalid Mahmoud

**Affiliations:** Chemistry Department, Faculty of Science, Beni-Suef University Beni-Suef Egypt mahabadawy36@gmail.com radwaraft@yahoo.com; Materials Science and Nanotechnology Dept., Faculty of Postgraduate Studies for Advanced Sciences (PSAS), Beni-Suef University Beni-Suef Egypt ahmed.elshahawy@psas.bsu.edu.eg Ahmedfarghali74@yahoo.com +20 226798209; Pharmacology Department, Faculty of Veterinary Medicine, Beni-Suef University 62511 Beni-Suef Egypt fa.pharma@yahoo.com Zienab_ryad@yahoo.com

## Abstract

Zn–Al layered double hydroxides (LDHs) were synthesized by a chemical method, while polyvinyl alcohol (PVA) nanofibers were fabricated by an electrospinning approach; we also synthesized Zn–Al LDH/cefotaxime (cefotax), Zn–Al LDH@PVA, and Zn–Al LDH/cefotax@PVA (LCP). Characterizations were performed by X-ray diffraction, Fourier transform infrared spectroscopy, field emission scanning electron microscopy, high-resolution transmission electron microscopy, energy dispersive X-ray spectroscopy, Brunauer–Emmett–Teller analysis, thermogravimetric-differential thermal analysis techniques, dynamic light scattering, X ray-florescence, and carbon, hydrogen, and nitrogen (CHN) analyses. The adsorption isotherm of cefotax and its entrapment percentage, release, and kinetics were also investigated. The results confirmed the elemental constituents of the mentioned formulas, which exhibited different degrees of crystallinity and different morphologies. Besides, these formulas were tested *in vitro* as antimicrobial agents and applied *in vivo* against second-degree wound burns induced in rats' skin. The adsorption of cefotax occurred chemically, and the experimental data were fitted with different isotherm models, where the Freundlich and Toth models gave the best fits. The entrapment percentage in LDH/cefotax was 77.41% and in LDH/cefotax@PVA, it was 67.83%. The sustained release of cefotax from LDH and LCP was attainable; the release percentages were 89.31% and 81.55% in up to 12 h, respectively. The release kinetics of cefotax from LDH fitted well with first-order kinetics, while that for LCP was parabolic. The formulas showed uneven antimicrobial effects against Gram-positive and Gram-negative bacteria; the best effect was exhibited by Zn–Al LDH/cefotax@PVA due to its sustained release. Finally, investigating the possibility of using these formulas in the clinical setting should be considered.

## Abstract 2

This study succeeded to formulate, characterize, and investigate cefotax release and kinetics, and to compare cetofax with other known antibacterial agents.

## Graphical abstract

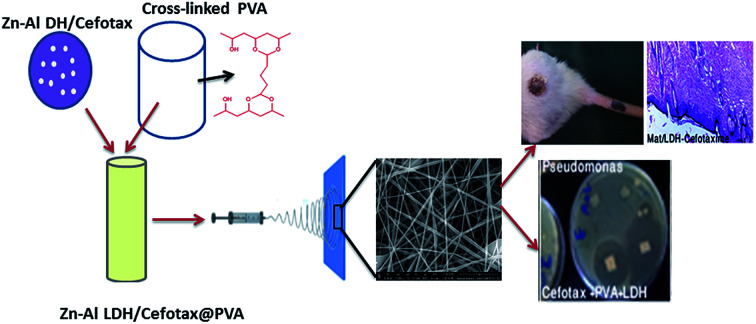

## 1. Background

Human skin is the most important and the largest organ of the human body.^1^ It acts as a barrier against the invasion of pathogens, such as bacteria and fungi colonization.^2,3^ Wounds to the skin are classified as acute, such as those due to burns, trauma, and abrasions, or chronic, such as the case with diabetic foot ulcers.^4^ In respect of burns and based on the burn depth, burns are divided into three degrees: superficial, partial, and full-thickness.^5^ In second degree burns, the regions of the skin affected are the epidermis and hypodermis;^6^ the healing process of this degree is intricate and multistage and comprises inflammation, proliferation, neovascularization, re-epithelialization, and wound contraction.^7,8^

It is essential to realize that burn wound infection is one of the factors that can lead to mortality and morbidity.^9^ Although the current antibiotics can substantially enhance the process of wound recovery, they cannot reach the infected area in their active form and at a sufficiently effective concentration; besides, they may incur some issues such as toxicity and resistance. Consequently, for antibiotics to be more efficient, their half-life should increase, and their dose above the minimum inhibition concentration of bacteria should be limited. It is well known that cefotax has significant antibacterial potential toward Gram-positive and Gram-negative bacteria^10^ and against various infections such as those of the respiratory tract, skin, joint, and bone.^11^ The short half-life of cefotax and the high resistance of β-lactamase against cefotax limit its use in clinical settings.^12^ To enhance the efficacy of cefotax, it is essential to control or to sustain its release at the site of infection.

Layered double hydroxides constitute a broad family of lamellar solids similar to anionic clay with a hydrotalcite structure.^13^ The structural formula of LDHs is (M^2+^_1−*x*_M^3+^_*x*_(OH)_2_)_*x*^+^_[A^*n*−^]_*x*/*n*_·*m*H_2_O, where M^2+^ is a divalent cation, M^3+^ is a trivalent cation, and A^*n*−^ is an intercalated anion.^14,15^ LDHs can intercalate biologically-active molecules through anion exchange,^16,17^ a property which has opened their applications in medicine and pharmacy^18^ as drug carriers for many antibiotics,^16,19^ anti-inflammatory agents,^20^ targeted drug delivery vehicles,^21–23^ and for wound healing.^24^ Recently, nanoparticles, beads, bandages, biofilms, and nanofibers have been considered intrinsic materials to attain sustained release.^25^ In particular, nanofibers are ideal candidates for wound dressing, because of their physical, mechanical, chemical properties, as well as high porosity, high surface to volume ratio, small pore size, and gas permeation properties.^26^ Besides, nanofibers are biocompatible, biodegradable, non-toxic, easily removed, and able to exchange gases and absorb excessed exudates of a wound.

Again, a primary concern of burn healing is the infection; in this respect, the target of the current study was to investigate the sustained release of cefotax and its kinetics against Gram-positive and Gram-negative bacterial strains; particularly, in the infection related to a second degree burn. The study succeeded through loading cefotax on two different nano-formulas: cefotax-loaded Zn–Al LDH and cefotax-loaded LDH@PVA. The novelty of cefotax-loaded LDH@PVA was manifested in the choosing of PVA, which is a synthetic water-soluble hydrophilic polymer, for the nanofiber formation, where the hydroxyl groups of PVA were cross-linked with the aldehyde groups chemically forming nanofibers with insoluble membranes, and forming hydrogen bonding with the hydroxyl groups of cefotax and the LDH. The high adsorption ability of LDH enhanced the entrapment efficiency and stability against light degradation, which maintained the release of cefotax; besides, Zn–Al hydrotalcite avoided skin reaction and supported removal of the inflammatory exudates. On the other hand, the LDHs are very sensitive when used as drug carrier in acidic media unless they are covered with a polymer film, which preserves them from degradation in acidic media; this is a field where new insights are still required and more research work needs to be undertaken.^20^

## 2. Materials and methods

### 2.1. Materials

Zinc chloride (ZnCl_2_) 97% was purchased from LOBA Chemie, India; aluminum chloride hexahydrate (AlCl_3_·6H_2_O) 98% from Alpha Chemika, India; hydrochloric acid (HCl) and sodium hydroxide (NaOH) 98% from Biochem for Laboratory Chemicals, Egypt. Cefotaxime sodium (C_16_H_16_NaN_5_O_7_S_2_) 100% purity (see Table 1)^27–30^ and 95% difloxacin were purchased from Pharma Swede pharmaceutical company. A tube of MEBO, Vaseline, Grotto ointment, was purchased from Julphar, Egypt; each tube contained (15 g). Glutaraldehyde (GA) and polyvinyl alcohol (PVA) with *M*_w_ = 115 000 kDa were purchased from Oxford Lab Chem, India, and bidistilled water was used throughout the experiment. All these chemicals were used without any further purification.

**Table 1:** Cefotax

Item		Reference
Common name	Cefotaxime	27
IUPAC name	(6*R*,7*R*)-7-[(*Z*)-2-(2-Amino-4-thiazolyl)-2-(methoxyimino) acetamide]-3-acetyl-oxymethyl-3-cephem-4-carboxylate	28
CAS number	64485-93-4	27
Molecular formula	C_16_H_16_N_5_NaO_7_S_2_	28
p*K*_a_	2.37, 3.03 and 4.21 from (COOH), amino thiazole and hydroxy triazinone respectively	30
Solubility	Freely soluble in water, slightly soluble in alcohol (absolute, 95%), insoluble in chloroform	29
Molecular weight (g mol^−1^)	477	28
Molecular structure	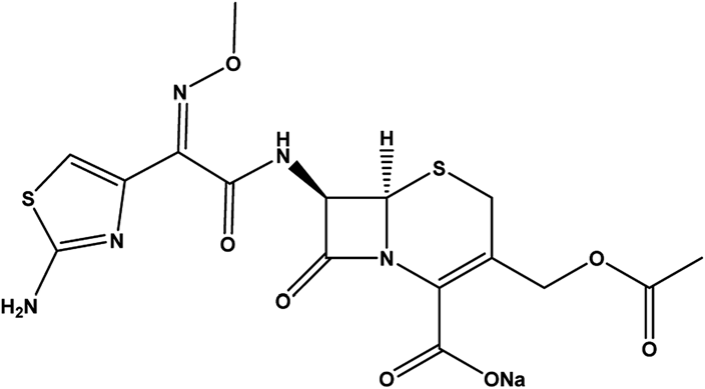	29

### 2.2. Synthesis of Zn–Al LDH and Zn–Al LDH/cefotax composite

Zn–Al LDH was prepared by the co-precipitation method as follows: a solution of 0.04 mol of ZnCl_2_ and 0.01 mol of AlCl_3_·6H_2_O (with a molar ratio of 4 : 1) was added to 100 mL bidistilled water with good mixing; then NaOH (1 mol L^−1^) was added dropwise until a complete white precipitate was obtained at pH 9. The resulting suspension lasted under vigorous stirring at 50 °C for 24 h, and the precipitate was collected, washed several times using bidistilled water and ethanol until pH 7, and finally dried at 40 °C for 24 h.^24^ The Zn–Al LDH/cefotax composite was synthesized by the aforementioned method with the same procedure. One difference was the ratio between the elemental constituents of the composite, whereby NaOH was added to a well-mixed solution of 0.04 mol of ZnCl_2_, 0.01 mol of AlCl_3_·6H_2_O, and 0.0006 mol of cefotax with a molar ration of 4 : 1 : 0.0625 ZnCl_2_ : AlCl_3_·6H_2_O : cefotax, respectively. Fig. S1a & b† display the elemental configuration of the prepared LDH and LDH/cefotax.

### 2.3. Synthesis of the polymeric cross-linked PVA nanofibers

A concentration (10 wt%) of the PVA water solution was prepared by stirring at 90 °C for 4 h. For the cross-linking reaction, 0.25% GA was added to the PVA solution under continuous stirring at 60 °C for 2 h. Then, the cross-linked polymeric solution was loaded into a plastic syringe (10 mL), and electrospun at an applied voltage of 18 KV to form the polymeric nanofibers. The solution was constantly supplied using a syringe pump at a flow rate of 0.7 mL h^−1^, and the spinning distance between the needle and the ground electrode was 14 cm.^31^

### 2.4. Synthesis of Zn–Al LDH@PVA and Zn–Al LDH/cefotax @PVA nanofibers

**Scheme 1:**
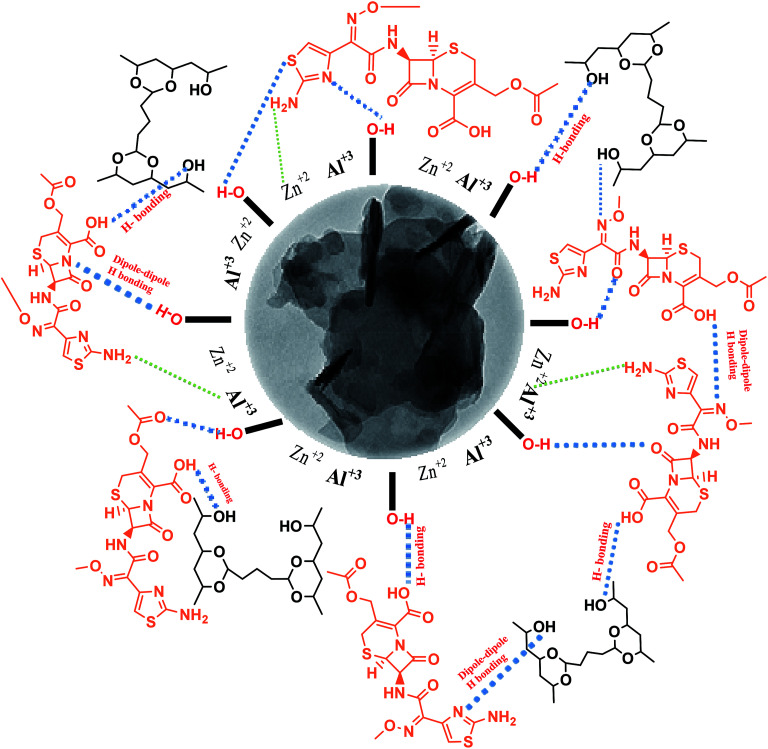
The idea involved loading cefotax on two different nanoformulas: cefotax-loaded Zn–Al LDH and cefotax-loaded LDH@PVA nanofibers to sustain its release at the site of infection. Rearrangement of Zn/Al ions, and partial replacement of chloride ions by cefotax molecules or by the adsorption of cefotax molecules on the surface of LDH occurred *via* hydrogen bonding; in light of the mentioned, it can be said that the likelihood of cefotax loading and even the partial intercalation between LDH, PVA, and cefotax was evidenced. The slow release of cefotax was achieved by the restriction of cefotax between LDH and PVA by hydrogen bond, dipole–dipole interaction, as well electrostatic force. The formulas were tested *in vitro* as antimicrobial agents, and applied *in vivo* against second degree wound burns induced in rats' skin.

For the Zn–Al LDH@PVA nanofibers, 0.1 g of the prepared Zn–Al LDH was added into 10 mL of the PVA cross-linked viscous solution with continuous stirring at 50 °C for 1 h, and the electrospinning procedures were conducted as mentioned. For the Zn–Al LDH/cefotax @PVA nanofibers, the same process as outlined in Scheme 1 was performed.^32^

### 2.5. Characterization study

The prepared materials were characterized as follow: the crystallinity was analyzed using an X-ray diffraction (XRD) system (PANalytical Empyrean, 202964, Sweden) with Cu Kα radiation (*λ* = 0.154 cm^−1^), at an accelerating voltage of 40 kV, current of 35 mA, scan angle of 5–80° and with a scan step of 0.04°. Analysis of the functional groups was performed utilizing a Fourier transform infrared (FTIR) spectroscopy system (Bruker-Vertex 70, KBr pellet technique, Germany), over the range of 400–4000 cm^−1^. The morphology and structure were analyzed by field emission scanning electron microscopy (FESEM, JEOL JSM-5900, Japan) and high-resolution transmission electron microscopy (H-RTEM JEOL JEM-2100, USA) with an acceleration voltage of 200 KV.

The elemental analysis of Zn–Al LDH was performed by energy dispersive X-ray spectroscopy (EDX, Quanta FEG-250, Germany), while the composition of Zn–Al LDH/cefotax was determined using elemental CHNS analysis (Vario, EL III analyzer), and X ray-florescence spectroscopy (XRF). The thermal stability was assessed by thermogravimetric differential thermal analysis (TGA-DTA) (Shimadzu Co. Tokyo, Japan); here, a sample of 50 mg of the tested formula was heated up to 1000 °C at a rate of 20 °C min^−1^ under a dynamic N_2_ atmosphere. The specific surface areas, specific pore volume, and pore-size distribution were measured with N_2_ adsorption isotherms and an automatic surface analyzer (TriStar II 3020, Micromeritics, USA) according to the Brunauer–Emmett–Teller (BET) theory. The zeta potential and hydrodynamic diameter were determined by dynamic light scattering (DLS) with a zeta-sizer analyzer (Nano-ZS90, Malvern Instrument Ltd, United Kingdom).

### 2.6. Adsorption experiments and percentage entrapment efficiency

The adsorption isotherms experiments were performed in 250 mL conical flasks as follows: Serial dilutions of cefotax (30, 50, 100, 150, 200, 250 mg L^−1^) were prepared from a cefotax stock solution (250 ppm), where these concentrations represent the initial concentration of cefotax before adsorption (C_0_). Then, 0.1 g of Zn–Al LDH was added to a constant volume (50 mL) of each dilution at pH 5, which was adjusted using a pH meter (Adwa-AD1030) by adding 0.1 M HCl and 0.1 M NaOH. All the solutions were left for shaking on an orbital shaker (SK-0330-pro) at a speed of 200 rpm at room temperature for 24 h. Next, Zn–Al LDH- loaded with cefotax from each initial dilution was separated by filtering through a syringe filter with a 0.22 μm pore size (Millipore Millex-G, Hydrophilic PVDF), and the residual concentration of cefotax (where *C*_*t*_ is the concentration of cefotax after adsorption at time *t* min) was measured with a UV-Visible spectrophotometer (UV-2600, Shimadzu, Japan) at 250 nm.

The efficiency of cefotax adsorption on Zn–Al LDH was calculated according to the following eqn (1):1
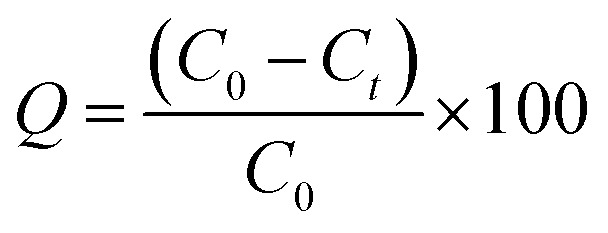
where *Q* is the adsorptivity (%), *C*_0_ is the initial concentration of cefotax in mg L^−1^, and *C*_*t*_ is the concentration of cefotax after adsorption at time *t* (min). The amount of the adsorbed cefotax at equilibrium *q*_e_ (mg g^−1^) was calculated through eqn (2):2
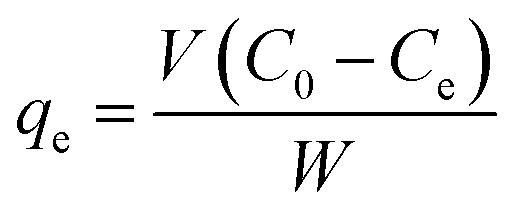
where *q*_e_ is the equilibrium adsorption capacity of the adsorbent by mg g^−1^, while *C*_e_ is the equilibrium concentration of cefotax according to the volume of the cefotax solution in liters, and *W* stands for the adsorbent (Zn–Al LDH) weight by the gram.

For calculation of the entrapped cefotax (% entrapment efficiency), the separated Zn–Al LDH-loaded with cefotax was centrifuged, and the concentration of the free cefotax (supernatant) was determined as mentioned. The percentage entrapment efficiency was calculated as follows according to eqn (3):3
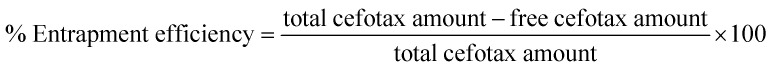


To ascertain the experimental reproducibility, all the experiments were conducted in triplicate, and the average concentration was determined using SPSS version 16. The mean and standard deviation (±SD) values were computed, and *p* values less than 0.05 were considered as statistically significant. All the preceding steps and equations involved use of the LCP formula.

### 2.7. Cefotax release and kinetics study

The cefotax release experiment of Zn–Al LDH/cefotax was conducted by monitoring the time-dependent release of cefotax molecules as follows: 1 mg of cefotax-loaded LDH (which contained the calculated adsorbed cefotax at equilibrium, eqn (2)) was dispersed in 10 mL of phosphate buffer saline (PBS) solution at pH 5.6 and 37 °C. Under continuous stirring, and at a constant interval time (100, 200, 300, 400, 500, 600 min), 1 mL PBS solution was withdrawn and replaced by 1 mL of fresh PBS to maintain the concentration gradient of the PBS solution. The concentration of the released cefotax within the withdrawn sample was determined as mentioned. The experiment was conducted in triplicate, and the average concentration was determined. To understand the cefotax release kinetics, the cefotax release profile was fitted according to the first-order and parabolic diffusion kinetic models. Again, all the preceding steps involved use of the LCP formula.

### 2.8. Animal preparation and burn induction

The Institutional Animal Care and Committee of Beni-Suef University, Egypt, approved all the conducted procedures. All the animal handling, study procedures, weighting, dosing, and burns induction and treatment were approved according to the Guidelines for Care and Use of Laboratory Animals of the Faculty of Veterinary Medicine, Beni-Suef University, also according to the Institutional Animal Care and Ethical Committee of Beni-Suef University, Egypt. Burn induction and healing activity were as follows,^33^ in brief, a total of 30 male albino rats (350–400 g, 6–8 weeks old) were anesthetized through an intraperitoneal injection of ketamine 5% (90 mg kg^−1^ B wt), and xylazine hydrochloride 2% (5 mg kg^−1^ B wt), and then a surgical incision of (20 × 20 mm) was induced on the back shaved area of the mice (20 × 20 mm). Next, a stainless-steel, cone-shaped device with a diameter of 2 cm and weighing 400 g was heated to 100 °C and placed locally for 5 s.

#### 2.8.1. Experimental design

According to the appropriate hygienic and regulated conditions, and after 2 h of burn induction, all the tested formulas were prepared in a dosage form of an ointment of vaseline and a fiber-based formulation with the concentration of 10% and were applied topically on the burned area once daily until complete healing occurred. According to the assessed formula, the rats were divided into six categorizes as follows: G1 (Mat/LDH-Cefotaxime MLC), G2 (Mat/LDH), G3, (Mat only), G4 (Grotto ointment), G5 (Standard MEBO ointment), G6 (Control negative-Vaseline only).

All the rats were monitored daily, and any burn fluid or evidence of infection or other abnormalities were noted and recorded. The burn-healing activity was assessed by the burn contraction percentage and closure time. The burn size was measured at 4, 8, 12, 16, and 21 days post-operation, and the burn-healing percentage was calculated by the Walker formula^34^ as per eqn (4):4
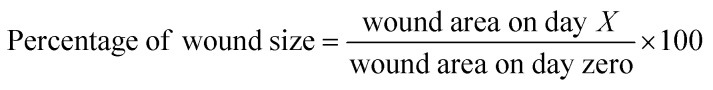


#### 2.8.2. Wound healing evaluation

The wound healing was checked daily for three weeks by assessing for macroscopic signs of inflammation (hotness, redness, swelling, and re-epithelization), an edema of about 4 mm, and according to the gross morphology of the wounds in terms of color and margin, besides necrosis of the epithelial and connective tissues. Further, assessment was made through ruler-measurement of the wound size immediately after burn infliction every four days till day 21.

Moreover, microscopic assessment by histopathology was performed. All the rats were euthanized at the end of the experiment using a mix of ketamine and xylazine with a 1 : 1 ratio at a dose of 0.1 mL/100 g. The sample specimens were excised from the deepest area of the burn and were preserved in 10% formalin for 72 h at room temperature. For the histopathology, 4 μm thick serial sections were cut using a rotary microtome (Microm HM350) equipped with a waterfall-based section transfer system (STS, Microm). The sections were stained with hematoxylin & eosin and investigated. The condition of the epidermis, dermis, and subcutaneous fat and skeletal muscle were taken into account for evaluation.

#### 2.8.3. Antibacterial study

The antimicrobial activity of the aforementioned synthesized materials was tested *versus* strains of Gram-positive bacteria (*S. aureus*, ATCC 25913) and (*Bacillus* ATCC 6633) and Gram-negative bacteria (*Pseudomonas* ATCC 27853) and (*E. coli*, ATCC 25922). The inhibition zone diameters of all the tested bacteria were determined by the disc diffusion method; this method was done according to CLSI guidelines. In this context, sterilized discs of PVA-tested material with different sizes (1, 0.5, 0.25, 0,06, and 0.125 mm) were obtained and were placed on separate sterilized Mueller–Hinton agar plates; each plate comprised a specific and aseptically diffused strain of the mentioned bacteria. Then, all the plates were incubated at 37 °C for 24 h. All the readings of the inhibition zones were taken in triplicate. In the same way, all the procedures were repeated with LDH@PVA, LDH/cefotax@PVA, cefotax, LDH/cefotax, and LDH. It is worth mentioning that the antibacterial activity of these materials was compared with a purity of 95% of the difloxacin standard drug (1000 μg mL^−1^).

Further, the broth dilution method was implemented to estimate the minimum inhibitory concentration (MIC) of the cefotax, and of the synthesized nanomaterial Zn–Al LDH/cefotax in the presence of both a positive control (cefotax or Zn–Al LDH/cefotax without bacterial culture) and negative control (bacterial cell culture without cefotax or Zn–Al LDH/cefotax). For the MIC study, a specific bacterial colony count through matching with a McFarland standard opacity tube of 10^8^ CFU mL^−1^ was obtained using sterile physiological saline, as previously mentioned.^35^ For each bacteria tested, a suspension of McFarland 100 μL (10^4^ CFU) in Muller–Hinton broth media was placed in different sterilized tubes containing a twofold serial dilution of the tested material (cefotax and Zn–Al LDH/cefotax). The results were recorded in terms of the MIC, which is the lowest concentration of the antimicrobial agent that inhibits growth. Equally important, the minimum bactericidal concentration (MBC) of those materials was detected. In the MBC experiments, a loopful from the MIC tubes of each tested material with two or more different concentrations of that material were placed on Muller–Hinton agar plates containing the tested bacteria. The concentration of the tested material that caused complete bactericidal inhibition of the bacterial growth after 24–48 h incubation at 37 °C was considered as the MBC.

## 3. Results and discussion

### 3.1. Material characterization

#### 3.1.1. X-ray diffraction

**Fig. 1:**
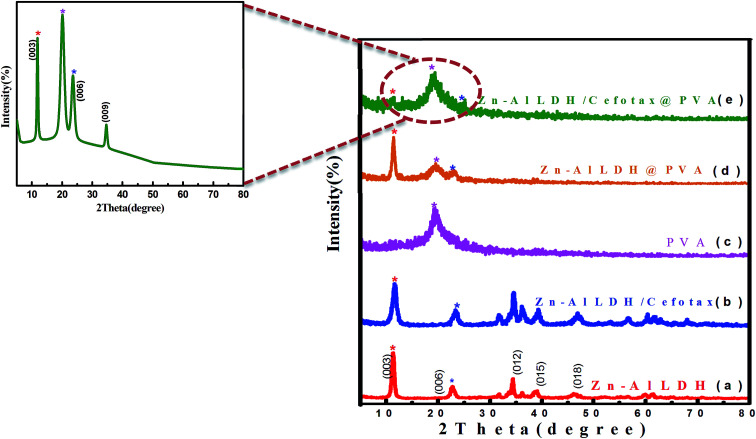
XRD patterns of the prepared materials.

Fig. 1 displays the XRD patterns of the formed materials. Overall, Fig. 1a indicates the unique characteristic peaks of a layered double hydroxide, either at a low 2*θ* angle with basal peaks at (003) and (006) or high 2*θ* angle at the (101), (015), (018), (012), (110) and (113) planes, in agreement with ref. 36. Especially, the peak of (003) was evidence of the basal reflection of the interlayer anion in the Zn–Al LDH material, since that peak positioned at 2*θ* = 11.31° may be attributed to the reflection of a hydroxyl (OH) or carbonate (CO_3_)^2−^ as a form of LDH.^37^ In addition, the peak cited at 36.2° revealed an additional phase of ZnO along with LDH.^38^ The image of the selected area electron diffraction pattern (SAED) shown in Fig. 3g matched well with the XRD data and confirmed the high crystallinity of the prepared LDH.

It was worth noting the similarity between the XRD result of Zn–Al LDH and that of Zn–Al LDH/cefotax shown in (Fig. 1b), in particular, the peaks marked with asterisks. However, some alterations were important to consider, for instance, the peak shifting to a high diffraction angle, the change in the relative intensity, and the broadening of the diffraction peaks. These alterations implied cefotax loading on LDH. Besides, the basal spacing of the (003) plane decreased from 7.82271 Å in the case of LDH to 7.58808 Å in Zn–Al LDH/cefotax, which revealed a less effective penetration of cefotax into the Zn–Al/LDH interlayers.^39–41^ This diminishing may refer to one of the following reasons: the removal of water molecules, rearrangement of Zn/Al ions, and partial replacement of chloride ions by cefotax molecules or the adsorption of cefotax molecules on the surface of LDH *via* hydrogen-bonding,^42^ as per the scheme submitted. Also, the d003 of the LDH and LDH/cefotax Nano composite was double that of d006 signifying a good layer structure.^43^Fig. 1c exhibits the main characteristic peak for PVA nanofibers centered at 2*θ* = 19.5°.^44,45^ It should be noted the obvious resemblance between the mentioned patterns and that of LDH@PVA (Fig. 1d), as well as the (Fig. 1e) LDH/cefotax@PVA nanohybrid, especially, the peaks at (003) and (006), which showed a good resemblance. In light of the aforementioned, it can be said that the likelihood of cefotax loading and even the partial intercalation between LDH, PVA, and cefotax was evidenced.^32,46^

#### 3.1.2. Fourier transformation infra-red spectroscopy

**Fig. 2:**
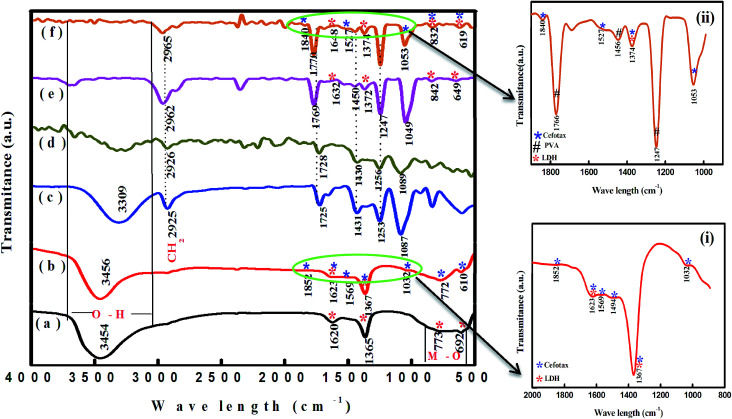
FTIR spectra of (a) Zn–Al LDH, (b) Zn–Al LDH/cefotax, (c) PVA, (d) cross-linked PVA, (e) Zn-AL LDH@PVA, and (f) Zn–Al LDH/cefotax@PVA.

It is important to recognize the structural alterations through the functional groups, and therefore we obtained the FTIR spectra, and these are presented in Fig. 2. Fig. 2, spectrum a shows the FTIR spectrum of Zn–Al LDH, where the broad peak at 3454 cm^−1^ is attributed to O–H vibrations of H_2_O molecules and hydrogen bonding in –OH interlayer groups and a bending vibration at 1620 cm^−1^.^47,48^ The intense peak at 1365 cm^−1^ reflected the symmetric and asymmetric stretching vibrations of a carbonate (CO_3_)^2−^, which may be formed during the sample preparation process.^37^ The peaks that appeared below 700 cm^−1^ belonged to M–O bond stretching and brucite lattice bending.^49^

Regarding LDH/cefotax, Fig. 2, spectrum b and inset (i) show the appearance of important bands of cefotax in the spectra of the Zn–Al LDH/cefotax composite as follows: stretching of O–H groups appeared at 3456 cm^−1^, N–H stretching is shown around 1569 cm^−1^, C=O stretching vibration of (COO)^2−^ at 1852.76 cm^−1^, the peaks at 1623, 1494.10, 1367.96, 1032, 772, and 610 cm^−1^ are due to the presence of C=O of an amide group, N–H, C=O, C–N, C–O, CH_2_, and C–S groups, respectively.^50^ Two clear pieces of evidence demonstrated the successful loading of cefotax on the Zn–Al LDH host: the appearance of cefotax bands and the difference between the two spectra, which was obvious and manifested in peaks shifting toward high wavenumbers, including the –OH group peak at 3454 cm^−1^ shifted to 3456.87 cm^−1^, the bending vibration of OH peak at 1620 cm^−1^ shifted to 1623.04 cm^−1^ denoting the promotion of hydrogen bonding interactions between Zn–Al LDH and cefotax,^24^ C=O at 1365.67 cm^−1^ shifted to 1367.96 cm^−1^, and the M–O bond stretching at 692 shifted to 610 cm^−1^.

In respect of PVA, Fig. 2, spectrum c exposed a broad and characteristic band of PVA at 3309 cm^−1^, while the peaks at 2925 and 1087 cm^−1^ were related to symmetrical and asymmetrical vibrations corresponding to CH_2_.^51–53^ It is important to be aware that adding GA as a cross-linker formed an acetal bridge between the polymer (PVA) and GA, which in turn decreased the intensity of the PVA OH group intensity in Fig. 2 in spectra d, e, and f.^54^ Besides, Fig. 2, spectrum e revealed an increase in the intensity of C=O of PVA and a shift of the CH_2_ peak from 2926 to 2962 cm^−1^, which may be attributed to the interaction between PVA, GA, and LDH.

Concerning, Zn–Al LDH/cefotax @PVA (LCP), Fig. 2, spectrum f and inset (ii) exhibited the characteristic bands of Zn–Al LDH, Zn–Al LDH/cefotax, and PVA, demonstrating the configuration of the nanocomposite. Again, the observed variance among the FTIR spectra signalized the interaction between the synthesized materials, which matches with the XRD data.

In spite of its low binding energy, the great importance of the hydrogen bond stems from its effects on the physical and chemical properties of the material, and is related to the degree of crystallinity, the regularity of the crystal system, and the amount of adsorbed water molecules bound.^55,56^ In general, a decrease in the intensity or a disappearance of a band involved in the H-bond interactions means that this interaction is an ‘intermolecular’ one, and *vice versa* for ‘intramolecular’ interactions. The formation of intramolecular hydrogen bonding did not show any shift in absorption upon dilution, while the intermolecular did. Consequently, from the overview of spectra a–f in Fig. 2, the FTIR study evidenced the intramolecular hydrogen bonding among the LDH/cefotax/PVA entities, as per Scheme 1. The calculated hydrogen bond intensity was the ratio of the absorbance bands at 3454 and 3456 cm^−1^ (for the –OH peak) and 1365.67 and 1367.69 cm^−1^ (for the C=O peak) in Zn–Al LDH and Zn–Al LDH/cefotax respectively. The absorbance ratio showed an increase in the case of Zn–Al LDH/cefotax nanocomposite (0.9709) more than that of Zn–Al LDH (0.807), indicating the hydrogen bonding interaction between the LDH and the loaded cefotax.^42^

#### 3.1.3. Microscopic study FESEM and TEM

The FESEM study was crucial to indicate the morphology of the prepared materials.

**Fig. 3:**
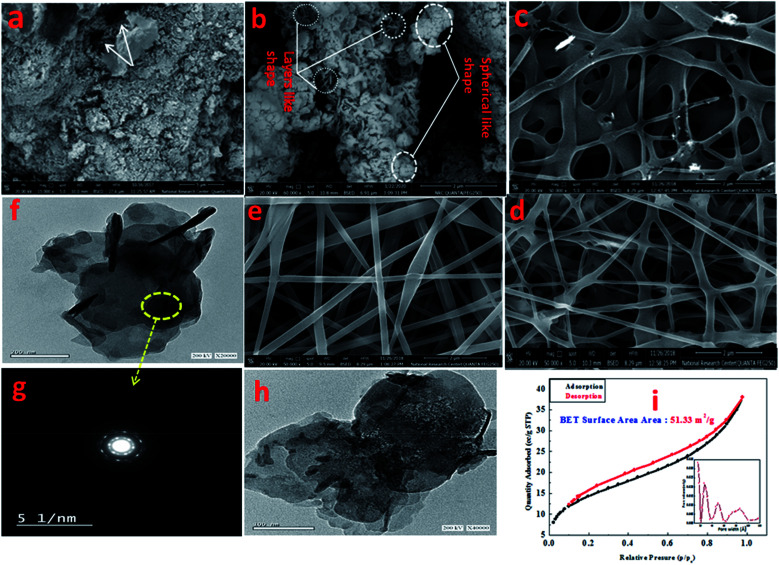
FESEM images of (a) Zn–Al LDH, (b) Zn–Al LDH/cefotax, (c) PVA, (d) Zn-AL LDH@PVA, (e) Zn–Al LDH/cefotax@PVA. TEM images of (f) Zn–Al LDH, SAED of Zn–Al LDH image (g), (h) Zn–Al LDH/cefotax and BET (surface area) of Zn–Al LDH/cefotax (i).

Fig. 3a presents the FESEM image of Zn–Al LDH, revealing accumulated layers in a form of a lamellar or sheet structure, thus reflecting the good preparation of LDH, which was in good agreement with ref. 57 and 58. Further, Fig. 3a shows the pore distribution (white arrow) with various volumes and sizes. Fig. 3b illustrates the FESEM image of Zn–Al LDH/cefotax with a spherical and lamellar morphology. The formed PVA nanofibers (Fig. 3c) seemed to be cross-linked with a thick, smooth, and uniform surface, as well occurring in random directions without any aggregation, implying that the factors in the electrospinning method were well-controlled during the preparation, which gave results consistent with previous results.^59,60^Fig. 3d exhibits the FESEM image of Zn–Al LDH@PVA nanofibers with decreased diameters (99.49 nm) compared with the diameters of the PVA nanofibers (124.1 mm) in Fig. S2.† The reasons attributed to the good dispersion of LDH in the PVA polymer solution include the change in solution electrical conductivity, viscosity, and surface tension, as reported also in the literature.^61,62^ Consequently, the condensation of the Zn–Al LDH@PVA matrix was low compared with that of PVA, as shown in Fig. 3d. The acquired images resembled those given in ref. 63 and 64. Fig. 3e shows a compacted structure of the fibers with uniform diameters, as well as a uniform distribution of LDH/cefotax over the nanofibers surface, either inner or outer surface. The justification for this was the good hydrogen bonding interactions among the (–OH) groups of LDH, cefotax, and PVA, as per Scheme 1 imagined. These interactions and inorganic filling increased the viscosity and viscoelastic force, which affected the surface tension of the PVA solution and hampered it being electrospun.^65^ On the other hand, one can notice the obvious difference between the TEM image of the LDH displayed in Fig. 3f and that of LDH/cefotax in Fig. 3h. This divergence, especially, the visible roughness of the LDH/cefotax surface, referred to the interaction between LDH and cefotax.

#### 3.1.4. Elemental composition, EDX, XRF, CHNS, and zeta potential analyses

It is worth highlighting that, to indicate the interaction between LDH and cefotax, it was necessary to identify the elemental constituents of the two entities. EDX analysis has now become common practice and is so practical that it is an essential part of SEM. The atomic weight percentages of all the samples were obtained from the elemental analysis. Fig. S1† displays the EDX spectra of: (a) Zn–Al LDH and (b) Zn–Al LDH/cefotax, indicating qualitative and quantitative characteristic peaks corresponding to all the different initial elements. The molecular formula as calculated was Zn_0.9_Al_0.23_(OH)_0.076_(cefotax)_0.004_(H_2_O)_0.097_Cl_0.24_, which matched with the XRF and thermal analysis, as reported in Table 2.

**Table 2:** D-spacing, surface parameters, and chemical compositions

Analytical method	wt%		Cefotax weight^b^ %	Zn–Al^a^ (mol mol^−1^)	Chemical composition
	Zn	Al			
XRF	43.276 (ZnO)	6.161 (Al_2_O_3_)	0.79	4 : 1	Zn_0.9_Al_0.23_(OH)_0.07_(cefotax)_0.004_(H_2_O)_0.09_Cl_0.04_

a Calculated from EDX data and XRF.

b Calculated CHNS analysis and TG-DTAdata.

Concerning the aqueous stability of the prepared materials, the average hydrodynamic particle sizes of the suspended solution of Zn–Al LDH and Zn–Al LDH/cefotax were 304.3 and 318.6 nm, respectively. The observed increase in the Zn–Al LDH/cefotax diameter was a result of the adsorption of cefotax on the LDH surface. Besides, the zeta potentials on the surface of Zn–Al LDH and Zn–Al LDH/cefotax were +28.4 and +15.9, respectively. The decrease in zeta potential value reflected that cefotax was extensively obtained as a successful nanocomposite formulation, not only through hydrogen bonding as referred by FTIR but also *via* electrostatic interaction.^24,66^

#### 3.1.5. BET and TGA-DTA analyses

The surface area of LDH and LDH/cefotax were determined by the BET method. Fig. S3† shows the surface area measurement of Zn–Al LDH (40.65 m^2^ g^−1^), whereby the pore diameter was determined using BJH analysis (0.061 nm) with a total pore volume of 0.056 cm^3^ g^−1^; here, there was a significant increase in the adsorption at a relative pressure of *p*/*p*_0_ > 0.02, which meant that the nitrogen uptake below *p*/*p*_0_ = 0.02 was negligible and the micropores were blocked, indicating that Zn-AL LDH was a type IV (mesoporous solid), which was attributed to the particle aggregation. Fig. 3i revealed that the adsorption/desorption process of LDH/cefotax belonged to hysteresis type-II; implying that the distribution of the pore size and shape was not well defined, and the materials were considered as macro-porous materials, whereby the surface area measurement of Zn–Al LDH/cefotax (51.33 m^2^ g^−1^) increased, which in turn increased the active sites of LDH and the amount of the adsorbed cefotax; and hence, this improved the cefotax efficiency at the infected sites. However, the total pore volume and the average pore diameter were decreased compared with the Zn–Al LDH.^67^ These results matched with the FESEM results for Zn–Al LDH/cefotax, where extra spherical-like shapes appeared beside layer-like shapes (Fig. 3b).

**Fig. 4:**
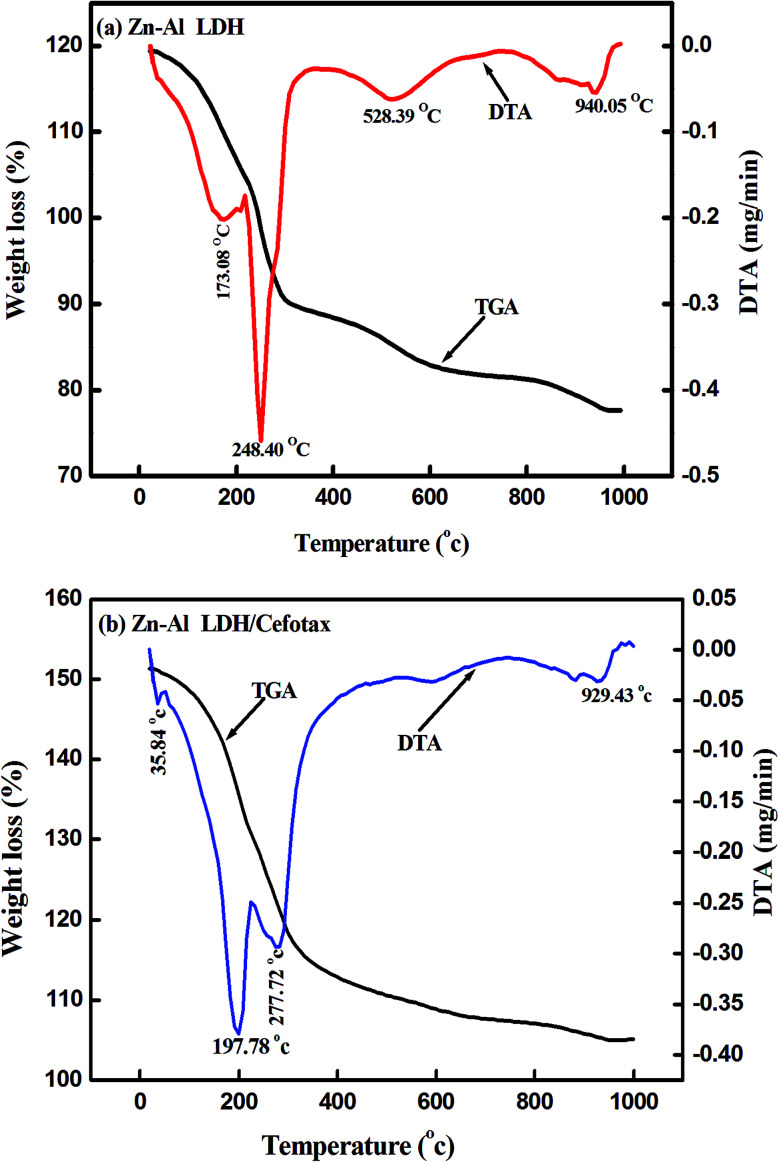
TGA-DTA of (a) Zn–Al LDH, (b) Zn–Al LDH/cefotax.

Fig. 4 shows the thermogravimetric analysis curves (TGA/DTA) for Zn–Al LDH and Zn–Al LDH/cefotax, which show the thermal decomposition behavior. In Fig. 4a, the first weight loss for Zn–Al LDH occurred from room temperature to 173.08 °C, corresponding to the adsorbed water (1.75 mg). A mass loss of 1.30 mg then took place in the temperature range to 248.40 °C, attributed to the dehydroxylation of the brucite-like layers.^68^ The decomposition of the brucite-like layer of chlorides occurred at 538.39 °C and the loss was 0.86 mg. The total weight lost at 970 °C was 41.20%.

As shown in Fig. 4b presenting the TGA/DTA curves for Zn–Al LDH/cefotax, the two consecutive endothermic peaks marked at 192 °C and 272.51 °C are related to the removal of the interlayered water and dehydroxylation. There were further mass losses around 608 °C and 929 °C in the TGA/DTA curves, which were due to the combustion of the organic guest cefotax. The decomposition started at 192 °C, suggesting the interaction of cefotax with Zn–Al LDH through electrostatic attraction, hydrogen bonding, and van der Waals force, which reflect how the thermal stability of the Zn–Al LDH-cefotax nanocomposite was enhanced. The thermal analysis matched the FTIR analysis results.

#### 3.1.6. Adsorption isotherm study

In the context of this research, an adsorption isotherm study was performed to describe the nature of the interaction between Zn–Al LDH and cefotax. The experimental data were fitted by various isotherm models, such as two-parameter isotherm models (Langmuir,^69^ Freundlich,^70^ Temkin^71^), and three-parameter isotherm models (Langmuir–Freundlich,^72^ Sips,^73^ Toth^74^). The parameters of the included models are reported in Table 3.

**Table 3:** Isotherm models and the measured parameters

Isotherm models	Expression	Adjustable model parameters	Values	*R* ^2^
**Two-parameters isotherm**				
Langmuir	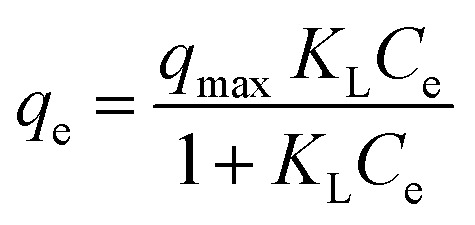	*q*_max_	52.47596	0.8262
		*K*_L_	184800.9	
Freundlich	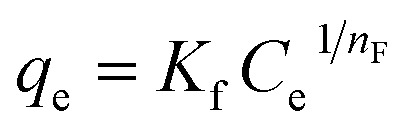	*K*_F_	1.458826	0.983389
		1/*n*_F_	0.85882	
Temkin	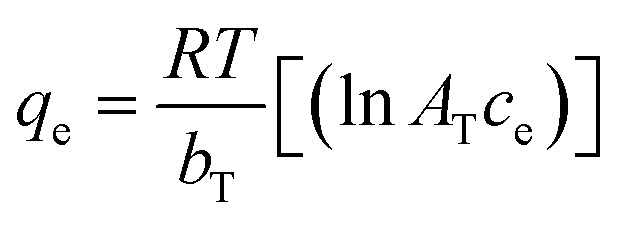	*b*_T_	184.3835766	0.89878
		*A*_T_	1.397952341	

**Three-parameters isotherm**				
Langmuir–Freundlich	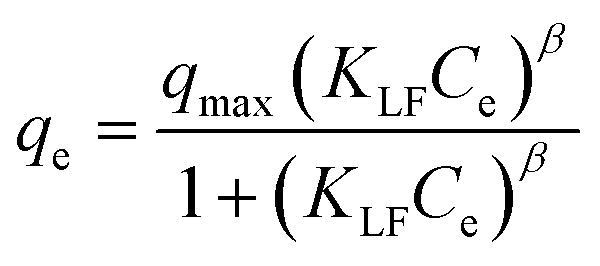	*q*_max_	12.62546693	0.899359
		*K*_LF_	0.125653135	
		*β*	1.426724324	
Sips	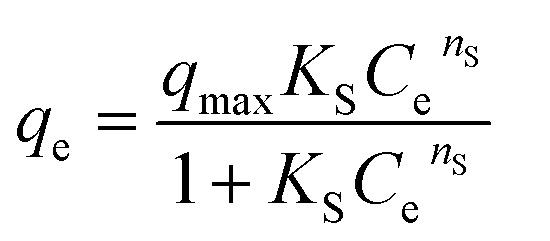	*q*_max_	19.83127	0.899359
		*K*_S_	0.033525775	
		*n*_S_	1.097429022	
Toth	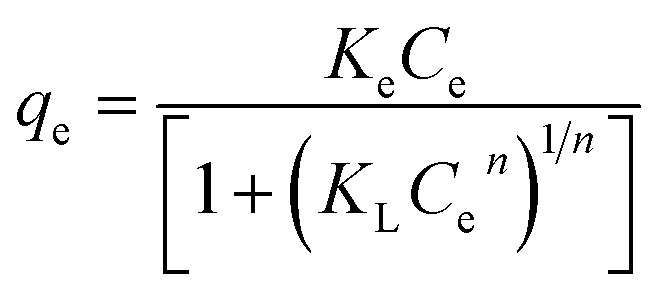	*K*_e_	2.3935047	0.98972
		*K*_L_	1.1554	
		*n*	2.56998058	

**Fig. 5:**
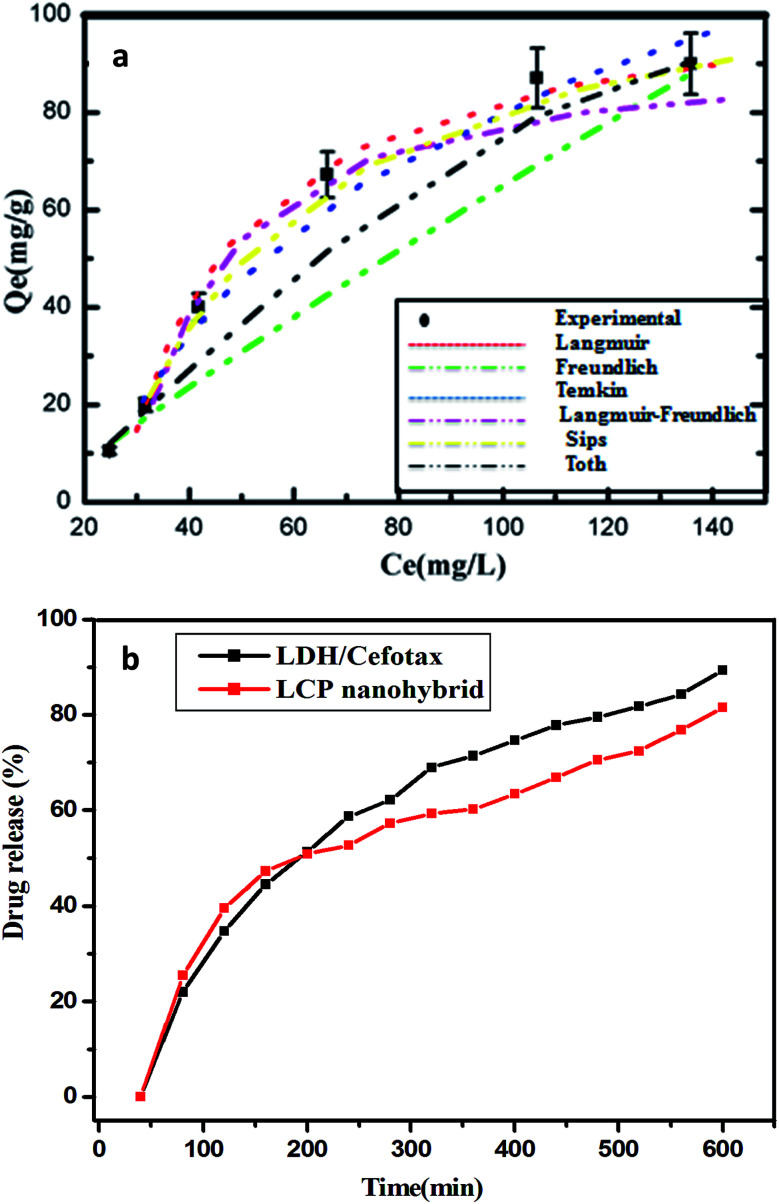
(a) Fitting the experimental data with isotherm models, error bars indicate the standard deviation of three experimental replications, (b) drug release of cefotax from LDH/cefotax and LCP nanohybride.

Fig. 5a exhibits the experimental adsorption isotherms and shows the equilibrium adsorbed amount of cefotax on Zn–Al LDH against cefotax equilibrium concentrations, with fitting the experimental data with the isotherm models. The error bars indicate the standard deviation of three experimental replications. Overall, it was evident that the acquired experimental data of the adsorption process could be fitted with the two- and three-parameters models. Freundlich and Toth models were the best fitting (*R*^2^ = 0.983389 and 0.98972, respectively), as reported in Table 3.

#### 3.1.7. Cefotax entrapment, release, and kinetics

Again, as aforementioned, the main limitation of cefotax in the clinical setting is the short half-life of cefotax, and so the main target of the current study was to sustain its release at the site of infection. As such, the current study considered the issues of drug load, release, and kinetics. The amount of the adsorbed cefotax at equilibrium *q*_max_ (mg g^−1^) was calculated and the results reported in Table 3 as 19.8 mg g^−1^, and the calculated percentage of cefotax entrapment was 77.41% in the case of LDH/cefotax, and 67.83% for LDH/cefotax@PVA, which are equivalent to 15.33 mg g^−1^, and 13.43 mg g^−1^ from the amount of the adsorbed cefotax at equilibrium (19.8 mg g^−1^), respectively.^75^ The decrease in the entrapment percentage of the LCP formula might be due to the loss of loosely bound cefotax on LDH (cefotax-LDH) during the formation of the LCP.^42^

In terms of cefotax release, Fig. 5b displays the percentage of released cefotax from LDH and LCP against the interval time. In total, a sustained release of cefotax from LDH and LCP was attainable. It was shown that, from the start point, the release percentage of cefotax from LDH increased steadily; until it was about 89.31% after 600 min from the start point. At the same time, while the percentage from LCP rose dramatically higher than LDH until the 150th minute, it then started to decrease, and equaled that of LDH at the 200th minute, then increased again consistently; however, it was still lower than LDH and reached 81.55% at the endpoint. The idea of sustaining the release of cefotax and the noted difference between LDH and LCP was significantly dependent on the structure of the formula; whereby cefotax bound on the LDH surface either by weak hydrogen bonds or electrostatic force, which gave the chance for fast release compared to LCP; whereas the slow release in the LCP formula resulted from the restriction of cefotax between LDH and PVA. Indeed, the success of the sustained release achieved a significant and evident effect to help avoid burn wound infection.

Now, a question is raised as to how is cefotax released in each formula, *i.e.*, LDH/cefotax, and LDH/cefotax@PVA? To answer this question, it was essential to study the release kinetics of the drug release rate using the first-order and parabolic models. Here, the drug-release profile of cefotax was fitted with the aforementioned equations.^42^5
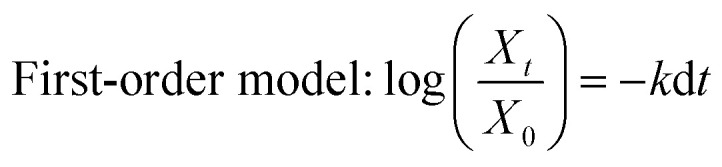

6 Parabolic diffusion mode (1 − *X*_*t*_/*X*_0_)/*t* = *k* − 1/2 + *a*

where, *X*_0_ and *X*_*t*_ are the amounts of drug present in the mentioned formulas matrix at a release time of 0 and *t*, respectively, while *k* and *k*_d_ are the overall release and apparent release constants. Mathew *et al.*^76^ stated that these models describe different physical processes. The first-order model describes a system where the release is dependent on the dissolution of the host material, while the parabolic model details a diffusion-controlled process.

**Fig. 6:**
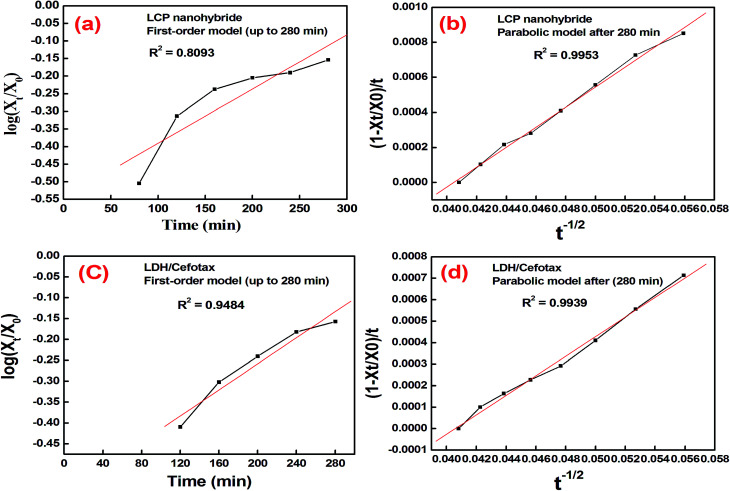
Time-dependent kinetic plots of the two models: first-order (a, c) and parabolic models (b, d) with the evaluated linear correlation coefficients (*R*^2^).

The acquired results are shown in Fig. 6, which displays the time-dependent kinetic plots of the two models with the evaluated linear correlation coefficients (*R*^2^). Fig. 6a represents the first-order model plot for LDH/cefotax@PVA with *R*^2^ = 0.8093, while Fig. 6b is the parabolic model plot with *R*^2^ = 0.9953. Fig. 6c presents the first-order model plot for LDH/cefotax (*R*^2^ = 0.9484), and Fig. 6d presents the parabolic model plot with *R*^2^ = 0.9939. Consequently, for LDH/cefotax@PVA, the release could be well fitted with the parabolic model, implying that the cefotax release was based on a diffusion method. For LDH/cefotax, the release fitted with the two models, but more with the parabolic, indicating that two physical processes could control the release, *i.e.*, diffusion with the parabolic model and dissolution of cefotax from the surface of LDH with the first-order model.

Overall, the presented results suggested that the release of cefotax from the LCP nanohybrid and Zn–Al LDH both involved dissolution up to 280 min, when most of the surface cefotax was dissolved in the medium solution and then the diffusion controlled model took over after 280 min. Cefotax diffusion occurs through intraparticle diffusion by anion exchange and by dissolution of the brucite layers. In addition, it is worth noting that the calculated kd of LDH/cefotax@PVA (525.56) was lower than that of LDH/cefotax (607.13), because of the compact and strong interactions between cefotax, LDH, and PVA in the LCP nanohybride, which in turn slowed and sustained the release, as shown in the accumulative release profile (Fig. 5b).

#### 3.1.8. *In vivo* experiments

##### 3.1.8.1. Body weight, wound healing activity, and histopathology

**Fig. 7:**
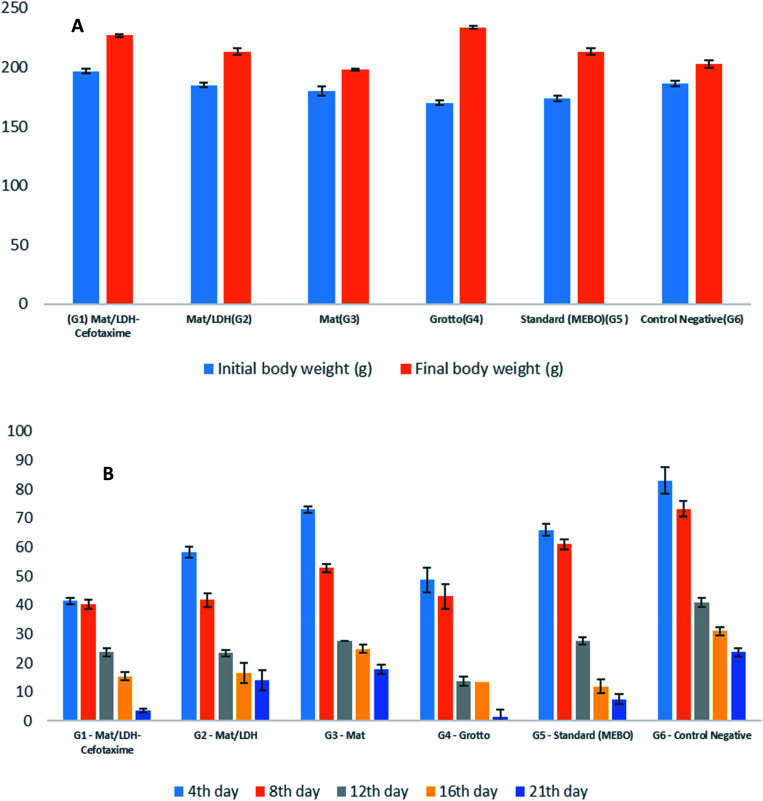
(A) Effects of different treatments on the rat's body weight. (B) Wound-healing activity of the burn by different nanomaterials in rats (*n* = 5).

Fig. 7A is a bar chart that displays the averages of the initial and the final averages of the body weights among the treatment groups. In general, a difference was noted but non-significant. In contrast, an evident divergence was observed in each group, in the order as follows: G4 (Grotto), G1 (Mat/LDH/Cefotax MLC), G5 (Standard MEBO ointment), G2 (Mat/LDH), G6 (Control negative – Vaseline only), and G3, (Mat only). All the treated animals showed no inflammation or signs of toxicity, as reported in Table 4. Fig. 7B is a chart presenting the wound sizes of the induced burns treated with different materials at different interval days (4, 8, 12, 16, and 21). The measurements of the experimental data are reported in Table 5. In total, the wound sizes differed among the groups along with the days of treatment. However, G4 (Grotto) was the best, followed by G1 (Mat/LDH/Cefotax), G2 (Mat/LDH), G5 standard (MEBO) ointment, G3 (MAT only), and G6 (control group).

**Table 4:** Effects of different treatments on the rat's body weight and signs of toxicity^a^

Group	Initial body weight (g)	Final body weight (g)	Weight gain (%)	Total deaths	Signs of toxicity
G1 Mat/LDH-cefotaxime	196.75 ± 2.1	226.75 ± 1.4	15.30	0	Nil
G2 Mat/LDH	185.00 ± 2.2	213.25 ± 2.5	15.13	0	Nil
G3 Mat	180.005 ± 3.7	198.00 ± 1.1	10	0	Nil
G4 Grotto	170.00 ± 1.7	233.75 ± 1.4	37.05	0	Nil
G5 standard (MEBO)	173.75 ± 2.3	213.00 ± 2.7	23.12	0	Nil
G6 control negative	186.25 ± 2.7	203.00 ± 3.2	9.13	0	Nil

a Values represent the mean ± SE of 5 animals in each group.

**Table 5:** Wound-healing activity of the burn by different nanomaterials in rats (*n* = 5)^a^

Group	Wound size (mm) and percentage of wound healing at					
	0 day	4th day	8th day	12th day	16th day	21st day
G1 Mat/LDH-cefotaxime	400.00 ± 0.0 (0%)	41.33 ± 1.2^cd^ (59%)	40.08 ± 1.6^c^ (60%)	23.75 ± 1.4^c^ (77%)	15.41 ± 1.3^c^ (85%)	3.58 ± 0.6^e^ (96.42%)
G2 Mat/LDH	400.00 ± 0.00 (0%)	58.25 ± 1.8^d^ (41.75%)	41.66 ± 2.3^cd^ (58.34%)	23.33 ± 1.2^c^ (87.67%)	16.50 ± 3.4^c^ (83.50%)	14.00 ± 3.4^c^ (86%)
G3 Mat	400.00 ± 0.00 (0%)	72.91 ± 1.2^b^ (27.1%)	52.75 ± 1.5^b^ (47.25%)	27.5 ± 0.0^b^ (72.5%)	24.9 ± 1.4^b^ (75.1%)	17.8 ± 1.7^b^ (82.2%)
G4 Grotto	400.00 ± 0.00 (0%)	48.66 ± 4.3^b^ (51.34%)	42.91 ± 4.4^c^ (57.09%)	13.75 ± 1.6^d^ (84%)	13.41 ± 2.4^d^ (95.67%)	1.5 ± 2.4^f^ (98.5%)
G5 standard (MEBO)	400.00 ± 0.00 (0%)	65.83 ± 2.07^c^ (34.17%)	61.00 ± 1.7^a^ (39%)	27.66 ± 1.3^b^ (72.34%)	11.91 ± 3.1^e^ (88.09%)	7.5 ± 1.7^d^ (92.5%)
G6 control negative	400.00 ± 0.00 (0%)	82.91 ± 4.6^a^ (17%)	73.16 ± 2.6^a^ (26.84%)	40.91 ± 1.5^a^ (59.09%)	30.91 ± 1.3^a^ (69.09%)	23.66 ± 1.3^a^ (76.34%)

a (a–e): degree of the significant.

**Fig. 8:**
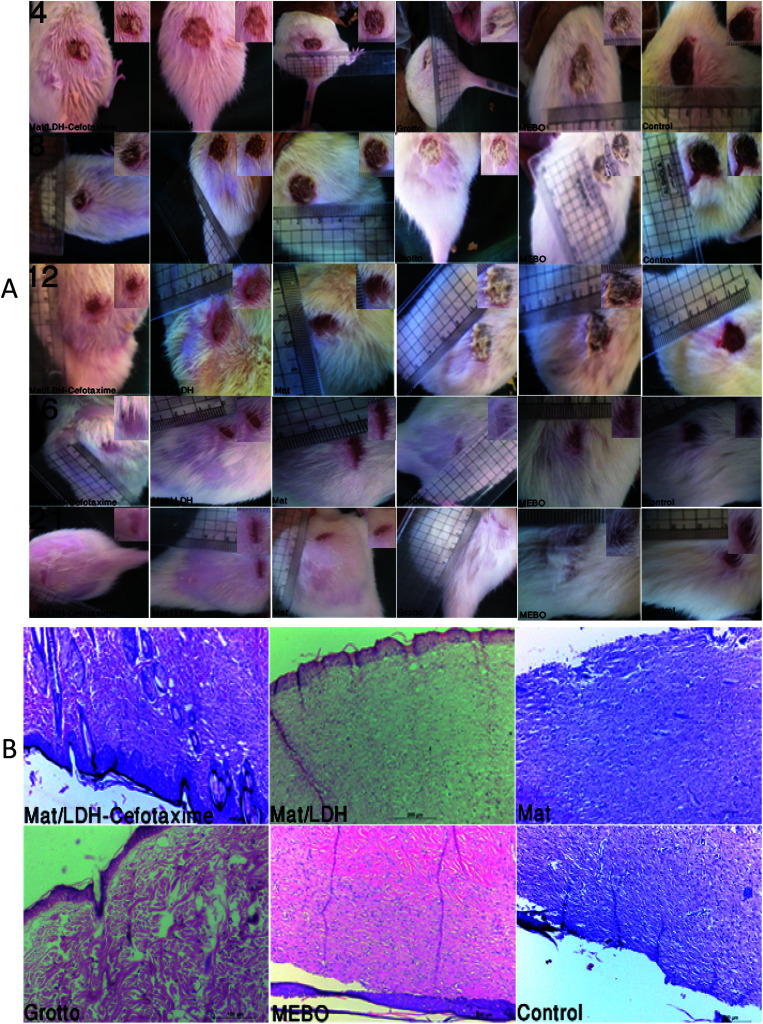
(A) Wound-healing activity of G1 (MAT, LDH-cefotaxime), G2 (MAT/LDH), G3 (MAT), G4 (Grotto), G5 (MEBO), G6 (Control Negative) in rats (*n* = 5), at zero, 4, 8, 12, 16 and 21 days of treatment, (B) histopathological investigation of the different treatments on the burnt skin.

The macroscopic and morphological alterations are demonstrated in Fig. 8A. Besides, Fig. 8B reveals the results from the histopathology. G4 (Grotto) and G1 (Mat/LDH-Cefotaxime) showed a complete healing of the epithelial layer with normal vasculature and cellular infiltration, while G2 (Mat/LDH) formed a normal epithelial layer with incomplete healing of the derm layer, and G3 (MAT only) and G6 (control group) exhibited discontinuity of the epidermal layer with granulation tissue in the derm layer, inflammatory cells with few capillaries, mild hemorrhage, and temperate necrotic foci; finally, the G5 standard (MEBO) ointment manifested incomplete formation of the epithelial layer and fixation on the derm layer.

It is important to realize that the antimicrobial activity of the tested materials is the key to wound healing with high efficiency, and to achieving this in a shorter time than normal. For instance, Grotto ointment contains important active ingredients, like vitamin E, bees wax, glycerin, allantoin, d-panthenol, lavender oil, and dimethicone. Fratini and co-workers [2016] mentioned that bees wax has antimicrobial activity.^77^ Likewise, in a major advanced study, Bano *et al.* demonstrated the antimicrobial effectiveness of PVA and its ability to facilitate the re-epithelialization of injured tissues, whereby the wound closure rate reached more than 96%.^78^ Additionally, an increasing number of studies [Koosha *et al.*, 2015; Sundaramurthi *et al.*, 2012] have found that the incorporation of PVA into nanofibers enhances the gene expression of fibroblasts cells and increases its viability besides improving the electrospun membranes biocompatibility.^79,80^ On the other hand, it has now been shown that zinc nanoparticles have antimicrobial activity; besides a role in improving collagen deposition, enhancing the angiogenesis, proliferating the fibroblast, and helping the fast closure of wounds.^81,82^

**Fig. 9:**
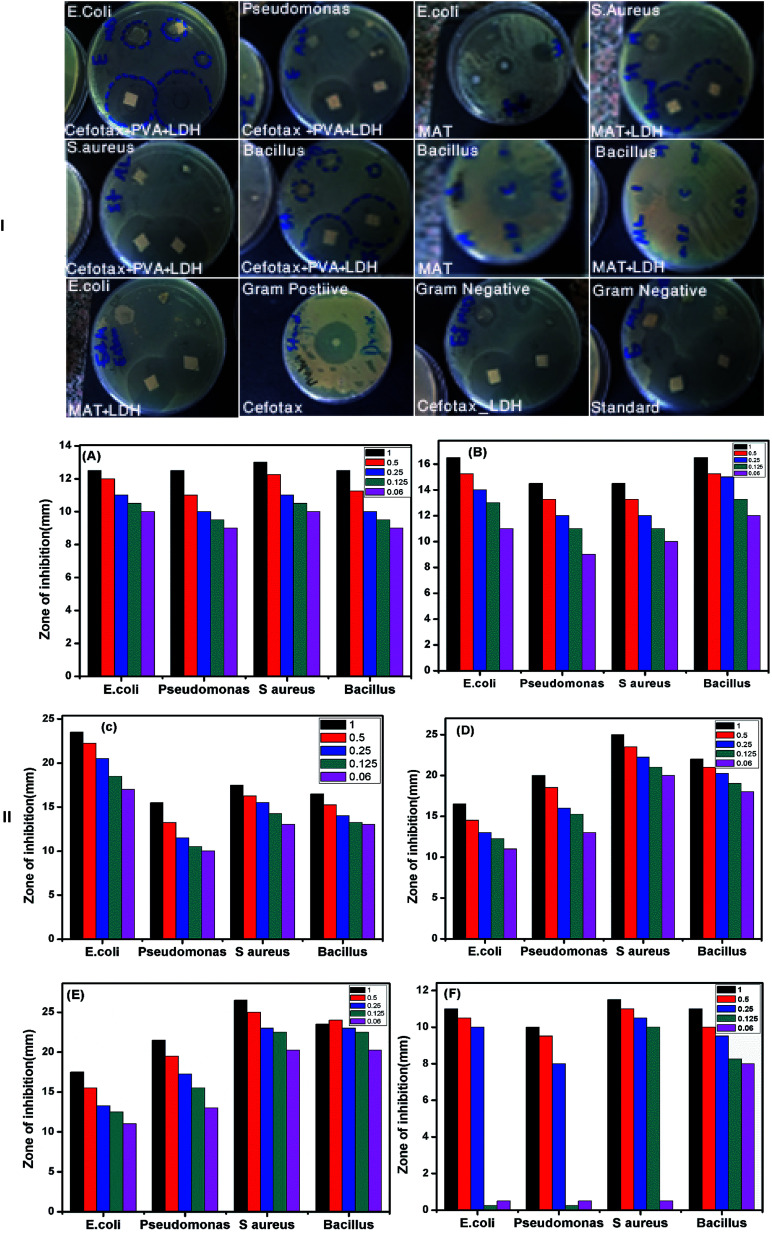
(I) Plates with zones of inhibition against different bacterial strains and at different sizes of the nanomaterials, (II) different zones of inhibition against different bacterial strains and at different sizes of the nanomaterials (A) PVA, (B) LDH@PVA, (C) LDH/cefotax@PVA, (D) cefotax, (E) LDH/cefotax, and (F) LDH.

In light of the mentioned aspects, the authors of the present study were encouraged to inspect the antimicrobial efficacy of the aforementioned materials. As mentioned above, the antimicrobial activity tests were conducted through MIC, MBC, and disc diffusion assay. Fig. 9I displays different plates with various strains of bacteria as Gram-positive and Gram-negative. In addition, the figure shows the inhibition zone of each strain with varying concentrations of the materials. The inhibition zone was measured in mm by the Agar diffusion method. As such, Fig. 9II is a bar chart illustrating the calculated mean of the inhibition zone (mm) on the *Y*-axis at different concentrations *versus* the diverse species of bacteria as mentioned above on the *X*-axis. Overall, the results reported that all the tested nanomaterials were effective against both Gram-positive and Gram-negative microorganisms, but the impact degree was different among the microorganisms.

As in the following examples, the diameter of the inhibition zone against Gram-negative bacteria was higher than against Gram-positive bacteria. The strongest activity was against *E. coli* (23.5 mm), followed by *S. aureus* (17.5 mm), *B. Subtilis* (17.5 mm), and *P. aeruginosa* (15.5 mm). It is important to highlight that, also the impact degree was different among the materials: Mat/LDH/cefotax showed excellent activity with the largest zone of inhibition followed by the LDH/cefotax, cefotax alone, and PVA as a Mat only, while Zn–Al LDH offered mild antimicrobial activity against both Gram-positive and Gram-negative bacteria.

**Fig. 10:**
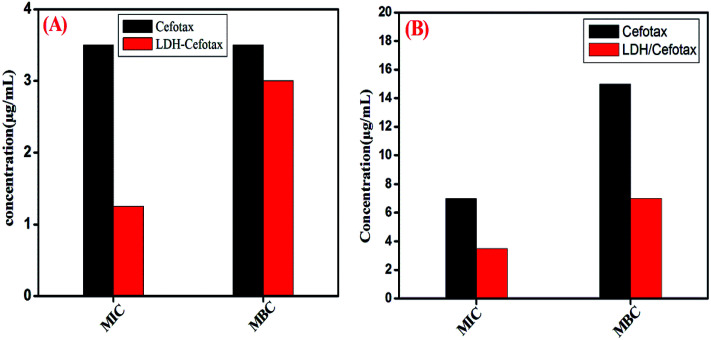
MIC and MBC of both cefotax and cefotax-LDH against (A) Gram positive, and (B) Gram negative bacteria.

It should be noted that the MIC and MBC of the investigated cefotax and cefotax-Zn/Al LDH *versus* Gram-positive and Gram-negative bacteria are shown in Fig. 10. Actually, the figure reveals that the recorded values of MIC and MBC of the cefotax and cefotax-Zn/Al LDH were significantly distinct from one species to another; aside from the MIC and MBC of the cefotax being higher than cefotax-Zn/Al LDH in both species. In addition, the significant difference in MIC between cefotax and cefotax-Zn/Al LDH was high in the Gram-positive bacteria compared to the Gram-negative bacteria, while the MBC was in contrast.

Indeed, destroying pathogenic microorganisms using antimicrobial agents is still a mysterious issue; because many factors affect the process, with the most important being the structure of the agent and that of the microbe. Cefotax is a third-generation cephalosporin family of medications and works by interfering with the bacteria's cell wall. Cefotaxime is a β-lactam antibiotic, which inhibits bacterial cell wall synthesis by binding to one or more of the penicillin-binding proteins (PBPs). This inhibits the final transpeptidation step of peptidoglycan synthesis in bacterial cell walls, thus inhibiting cell wall biosynthesis. The current study expected the formation of reactive oxygen species (ROS), which would be able to destroy the phospholipid layer of the cell membrane and damage DNA/RNA and proteins; the sources of the ROS were the metals of the LDH, whereby Zn in the LDH played an essential role in accelerating the wound healing.^83^

To conclude, it is worth remembering that burn wound infection is a crucial issue, and the clinical setting of cefotax is limited. The standing study succeeded to formulate, characterize, and investigate cefotax release and kinetics, and to compare cetofax among the aforementioned materials as antibacterial agents. On the whole, the formulas of LDH/cefotax and LDH/cefotax@PVA managed to achieve a sustained release of cefotax without any significant burst release at the site of infection and showed enhanced efficacy compared to the cefotax alone. For that reason, we can say that LDH and PVA nanofibers are good cefotax carriers with a high entrapment capacity and ability to sustain its release over considerable time. In closing, the study encourages investigating this formula in humans. Whatever the clinical stage of the patient or disease, with its use we will be able to maintain high constant levels of cefotax because of its improved pharmacokinetics behavior; and as a result, the dosage will be more easily improved, and the dosing intervals could be expanded.

## Funding

The present study was supported by individual funding.

## Consent for publication

The authors consent this manuscript for publication.

## Availability of data and material

The authors emphasize the availability of data and materials.

## Ethics approval and consent to participate

The authors followed the ethics of research, approved and consented to participate in this study.

## Conflicts of interest

The authors confirm no competing interests.

## Supplementary Material

RA-010-C9RA08355C-s001
