# Supplementary figures and images for: Chemisorption and sustained release of cefotaxime between a layered double hydroxide and polyvinyl alcohol nanofibers for enhanced efficacy against second degree burn wound infection
Source: RSC Adv. 2020 Apr 1;10(22):13196–214. doi: 10.1039/c9ra08355c (PMC9051420; doi:10.1039/c9ra08355c)

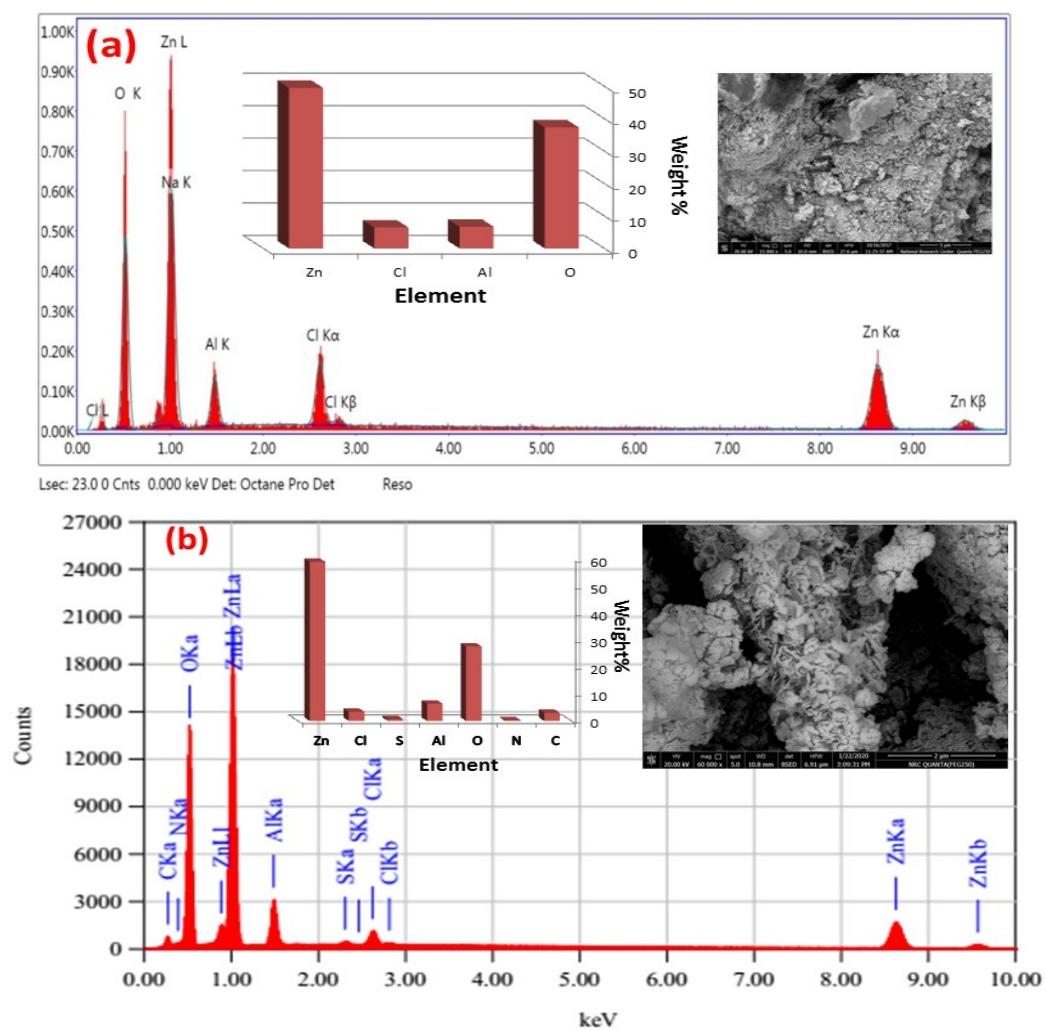

**Fig S1:** EDX of (a) Zn-Al LDH, (b) Zn-Al LDH/Cefotax.

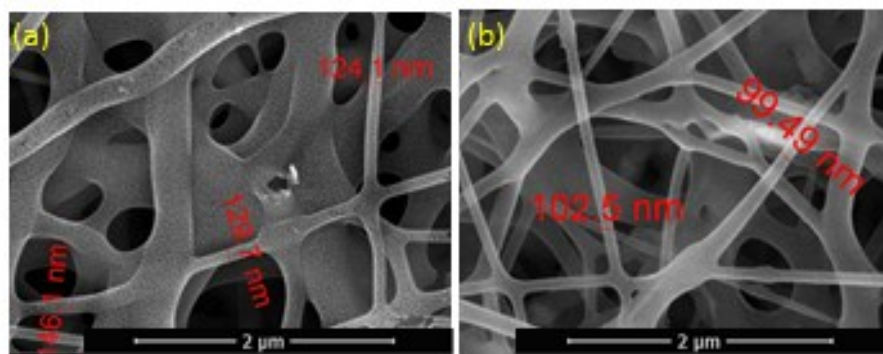

**Fig S2:** (a) Pristine PVA, (b) Zn-Al LDH@PVA.

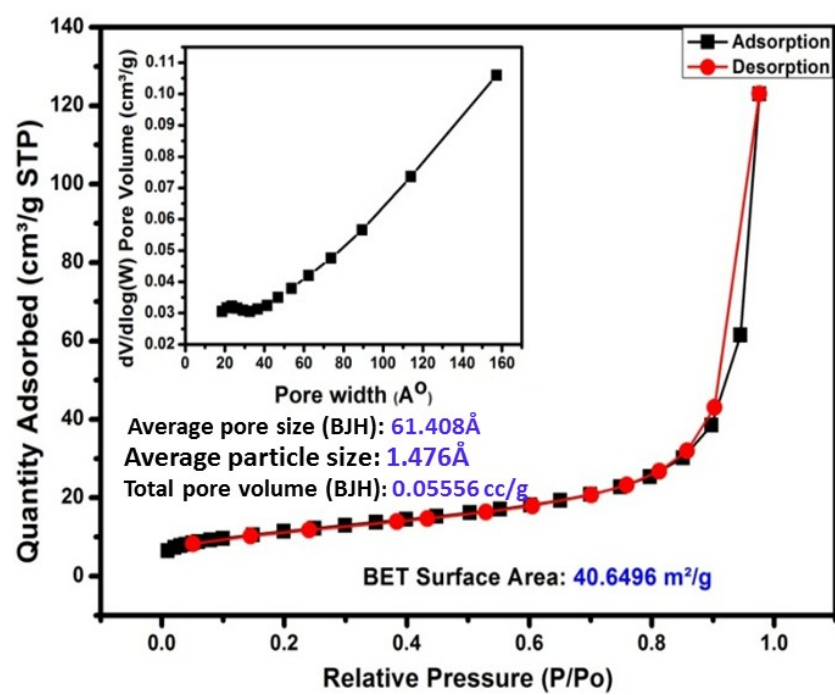

**Fig S3:** BET of Zn-Al LDH.

Supplement: RA-010-C9RA08355C-s001 [file RA-010-C9RA08355C-s001.pdf]
